# Aneurism of the Thoracic Aorta, with Death from Embolism of the Abdominal Aorta

**Published:** 1884-12

**Authors:** 


### Aneurism of the Thoracic Aorta, with Death from Embolism of the Abdominal Aorta.

A case of aneurism of the thoracic aorta, with death from embolism of the abdominal aorta, is reported in the *Lancet*, Aug. 30th, from the Liverpool Northern Hospital:

A. H., a sailor, aged 28, was admitted to the hospital the 29th of last March, complaining of pains in his joints and in front of chest. No physical signs of disease were at the time discovered. On April 12th a slight cough appeared, and on the 16th an area of dullness over upper part of sternum, continuous with the cardiac dullness, was first noticed; no bruit was then audible; the right radial pulse was a little smaller than the left.

On April 30th distinct pulsation could be felt over the upper part of the sternum and costal cartilages on the right side. A bruit was also now audible at the right second intercostal space and conducted into the subclavians. A sphygmographic tracing showed a curve with rather slow ascent and well-marked, rounded top. Infuell's treatment was adopted. By June 23d he had become tired of lying still and insisted on sitting up in bed. Dyspnœa, cyanosis and œdema of the face, with distension of the veins of the neck, marked the increase of intrathoracic obstruction. The pain in the chest was now so severe that he could not sleep without morphia.

On July 9th he was feeling rather easier than usual, but at noon he was seized with rather violent pain in the abdomen became greatly callapsed and bathed in a cold perspiration He complained of numbness in his legs and of inability to move them. No pulsation could be felt in either femoral artery, and both legs were cold and livid. At six o'clock the same evening he spat up some blood, and gradually sank, death occurring at 10.30 P. M.

*Necropsy*—Immediately above the attachment of the aortic valves the aorta was dilated, forming a large, irregular, but more or less spherical cavity, about the size of a fœtal head. The aneurism involved the whole of the artery, from its origin to the beginning of the descending portion. Its walls were very thin, and little laminated clots were found adherent to them; but the cavity contained a large quantity of soft, dark clot, in places indistinctly laminated. The remainder of the aorta was fairly healthy, but was completely filled with a soft, dark clot, terminating at its bifurcation. On cutting into the clot, no portion of different consistence was found in its interior, the whole clot resembled, very closely, that found in the aneurism. There was no other pathological change. The immediate cause of death was, no doubt, the detachment of a portion of the soft clot contained in the aneurism and its impaction in the bifurcation of the aorta, followed by secondary thrombosis filling nearly the whole vessel.

